# Case report: Targeted treatment strategies for Erdheim-Chester disease

**DOI:** 10.3389/fonc.2024.1305518

**Published:** 2024-03-14

**Authors:** Anita Gulyás, László Imre Pinczés, János Mátyus, Edit Végh, Judit Bedekovics, Judit Tóth, Sándor Barna, Zsolt Hunya, Imre Lőrinc Szabó, Annamária Gazdag, Árpád Illés, Ferenc Magyari

**Affiliations:** ^1^ Division of Hematology, Department of Internal Medicine, Faculty of Medicine, University of Debrecen, Debrecen, Hungary; ^2^ Doctoral School of Clinical Medicine, University of Debrecen, Debrecen, Hungary; ^3^ Division of Nephrology, Department of Internal Medicine, Faculty of Medicine, University of Debrecen, Debrecen, Hungary; ^4^ Division of Rheumatology, Department of Internal Medicine, Faculty of Medicine, University of Debrecen, Debrecen, Hungary; ^5^ Department of Pathology, Faculty of Medicine, University of Debrecen, Debrecen, Hungary; ^6^ Department of Oncology, Faculty of Medicine, University of Debrecen, Debrecen, Hungary; ^7^ Division of Nuclear Medicine and Translational Imaging Department of Medical Imaging, Faculty of Medicine, University of Debrecen, Debrecen, Hungary; ^8^ Department of Orthopaedics and Traumatology, Faculty of Medicine, University of Debrecen, Debrecen, Hungary; ^9^ Department of Dermatology, Faculty of Medicine, University of Debrecen, Debrecen, Hungary; ^10^ Division of Endocrinology, Department of Internal Medicine, Faculty of Medicine, University of Debrecen, Debrecen, Hungary

**Keywords:** Erdheim-Chester disease (ECD), histiocytosis, BRAF inhibitors, interferon alpha, cobimetinib, myeloid neoplasm, PET/CT

## Abstract

**Introduction:**

Erdheim-Chester disease (ECD) is a rare disease that belongs to the group of Dendritic and histiocytic neoplasms. Only 2000 cases have been reported worldwide. It can present with a wide range of symptoms, making a differential diagnosis especially difficult. The primary and most important diagnostic tool is a biopsy of the affected organ/tissue. Nowadays the analysis of different mutations affecting the BRAF and MAPK pathways makes it possible to use targeted treatments, such as vemurafenib, dabrafenib, or cobimetinib.

**Objective:**

Our aim is to present the results of three male patients treated in our hematology department.

**Results:**

Our BRAF mutation-positive patient presented with retroperitoneal tissue proliferation and diabetes insipidus. The initial therapy of choice was dabrafenib. After 3 months of treatment, ^18^F-fluoro-deoxyglucose positron emission tomography (FDG-PET)/computed tomography (CT) scans showed regression, and after 2 years of treatment, no disease activity was detected. In our second patient, a recurrent febrile state (not explained by other reasons) and diabetes insipidus suggested the diagnosis. A femoral bone biopsy confirmed BRAF-negative ECD. The first-line therapy was interferon-alpha. After 3 months of treatment, no response was observed on ^18^FDG-PET/CT, and treatment with cobimetinib was started. The control ^18^FDG-PET/CT imaging was negative. Our third patient was evaluated for dyspnea, and a CT scan showed fibrosis with hilar lymphadenomegaly. A lung biopsy confirmed BRAF-negative ECD. We started treatment with interferon-alpha, but unfortunately, no improvement was observed. Second-line treatment with cobimetinib resulted in a partial metabolic response (PMR) according to control ^18^FDG-PET/CT.

**Conclusions:**

Our results demonstrate that an appropriately chosen treatment can lead to a good therapeutic response, but dose reduction may be necessary due to side effects. With advanced targeted therapeutic treatment options, survival and quality of life are significantly improved.

## Introduction

Erdheim-Chester disease (ECD) is a rare histiocytic neoplasm, with only 2000 cases reported worldwide (1). ECD is the result of the clonal proliferation of myeloid progenitor cells, driven by the somatic mutations of BRAF and other members of the MAPK signaling pathway (2). Recently a genomic association in the 18q12.3 region has been identified for ECD. The closest gene to this region is SETBP1, which encodes a protein involved in myeloid cell proliferation. However no somatic mutations in SETBP1 have been reported in ECD, this gene has been associated with clonal hemoptoesis, which may play a role in the pathogenesis of ECD (3). According to the 2022 World Health Organization (WHO) Classification of Hematolymphoid Tumors it belongs to the group of Histiocytic neoplasms (4).

ECD is a multisystem disorder, with predominant involvement of the long bones, heart, blood vessels, lungs, central nervous system (CNS), kidneys, and skin (5–7). The diagnosis is based on characteristic clinical, laboratory, histological, and radiological findings (2, 7). Pathologic diagnosis is based on positivity for CD14, CD68, CD163, and Factor XIII and negativity for CD1a and Langerin (CD207) (1). The typical microscopic appearance is the presence of cellular proliferations with foamy cytoplasm and spongy bone (2). It is most common in middle-aged adults, and the male:female ratio is usually 3:1 (1). The initial evaluation should be performed according to consensus-based recommendations (2).

A small group of ECD patients are asymptomatic at the time of diagnosis, and regular follow-up is the priority in terms of treatment, compared to most patients who are symptomatic or have clear evidence of CNS involvement and require treatment as soon as possible (8). Mutational analysis plays an essential role in the selection of an appropriate therapy. Members of the RAF family (such as BRAF) are serine/threonine kinases that can activate MAP kinase (MEK 1/2) and extracellular signal-regulated kinases (ERK1/2). Both the phosphorylated targets of MEK 1/2 and ERK1/2 are involved in cell survival, proliferation, and differentiation. More than 50% of patients are positive for a BRAF mutation. BRAF V600 wild-type ECD lesions have been found to contain somatic mutations in the MAPK/ERK signaling pathway (9). In these cases, BRAF inhibitor therapy (vemurafenib, dabrafenib) is available under a compassionate use program. In patients who have progressed or failed to respond to BRAF inhibitors, (BRAFi) have wild-type BRAF, or harbor MEK pathway alterations, MEK inhibitor (MEKi) treatment (cobimetinib, trametinib) may be a reasonable option. MEKi therapy can be used regardless of MAPK mutations. A subset of patients with histiocytic neoplasms are positive for activating mutations upstream of RAS-RAF-MEK1/2, including in the receptor tyrosine kinase CSF1R, and can be successfully treated with a CSF1R inhibitor (10).

Other systemic approaches may be considered if targeted therapies are ineffective, such as interferon alpha, anakinra, tocilizumab, or mammalian target of rapamycin (mTOR) inhibitors (2, 11–14).

This publication presents the medical history and treatment results of three patients treated for ECD in our clinic.

## Case description

### Case 1

**Figure 1:**
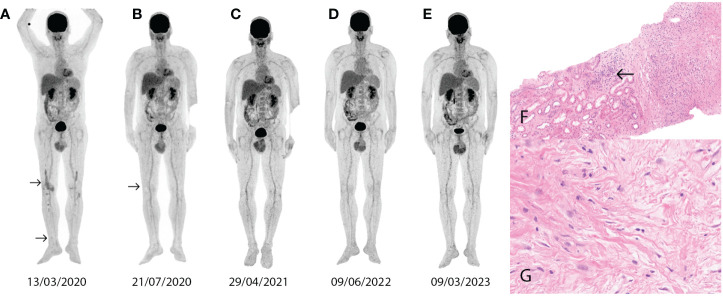
Abnormal contrast uptake (arrows) on the ^18^FDG-PET/CT image in the lower extremities (SUVmax 3.0) **(A)**. The control ^18^FDG-PET/CT image shows regression after 4 months of dabrafenib treatment (SUVmax 1.5) **(B)**. Control ^18^FDG-PET/CT images show CMR after dabrafenib treatment **(C–E)**. A perirenal tissue sample shows fibrosis, macrophages, and lymphoplasmocytic infiltration (arrow) (hematoxylin and eosin x100) **(F)**. Reactive inflammatory cells and fibrosis are seen in the perirenal tissue biopsy (hematoxylin eosin x400) **(G)**.

A 42-year-old male patient with a history of essential hypertension presented in 2019 with low back pain, polyuria, and polydipsia. An abdominal CT scan followed by an MRI (Magnetic Resonance Imaging) scan showed a “hairy kidney” characteristic of ECD. Laboratory tests showed elevated calcium and low potassium levels, liver, and kidney function tests were in the normal range, as were, lactate dehydrogenase (LDH) levels and inflammatory markers. Central diabetes insipidus was confirmed. In 2020, a perirenal tissue biopsy was performed, supporting the diagnosis of ECD (**Figures 1F, G**). In April 2020, mutational analysis confirmed the BRAF V600E mutation. ^18^FDG-PET/CT was performed and showed typical osteosclerotic lesions in the distal bones (**Figure 1A**). MRI of the brain showed no signs of CNS involvement and after a CT scan and echocardiography we found no evidence of cardiac involvement. The first-line treatment was dabrafenib, a BRAF inhibitor, initiated at a dose of 75 mg twice daily. We preferred dabrafenib over vemurafenib due to its slightly more favorable side-effect profile. In July 2020, the control ^18^FDG-PET/CT scan showed disease regression (**Figure 1B**), and subsequent control ^18^FDG-PET/CT scans indicated complete metabolic remission (CMR) (**Figures 1C–E**). In August 2023, the routine laboratory and imaging test results also supported CMR, with continued use of dabrafenib. In addition to radiological and laboratory findings, the patient experienced improvement in disease-related symptoms (such as bone pain).

### Case 2

A 55-year-old male patient with a history of ischemic heart disease, essential hypertension, and type 2 diabetes mellitus presented in 2016 with a recurrent fever of unknown origin. Laboratory evaluation revealed an elevated C-reactive protein (CRP) level. Results of kidney, and liver function tests were normal, as were blood levels of electrolytes, calcium, glucose, and LDH. Routine endoscopic and imaging studies for malignancy and immunologic screening tests were negative. The possibility of chronic cholecystitis of infectious origin was raised, for which a laparoscopic cholecystectomy was performed. Other extensive infectious disease work-ups were also negative.

**Figure 2:**
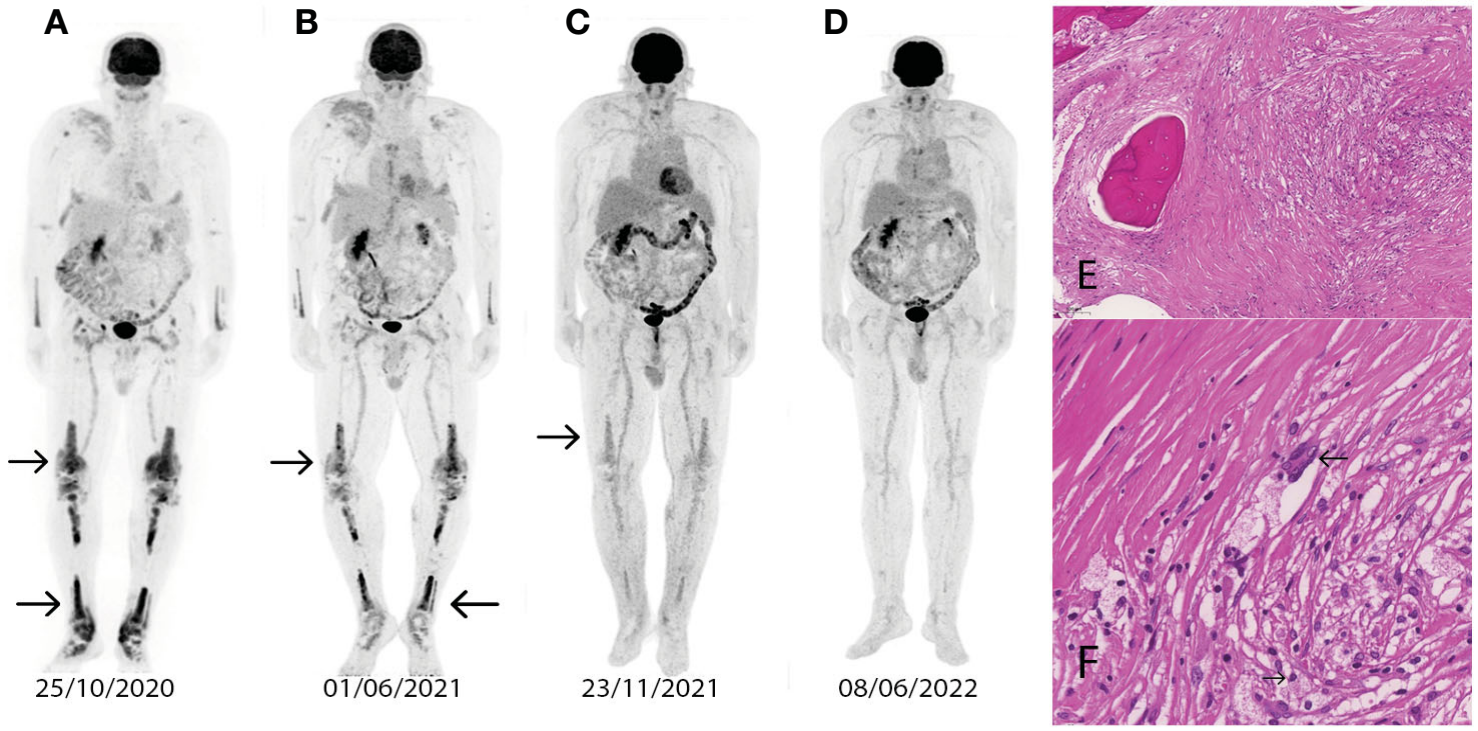
Abnormal contrast uptake (arrows) on the PET/CT image in the lower extremities (SUVmax 11.9-10.9) and at the pectoralis major (SUVmax 5.4) **(A)**. The control ^18^FDG-PET/CT image after interferon-alpha therapy shows refractory disease (SUVmax 18.8-16.0, 5.6-5.4) **(B)**. Control ^18^FDG-PET/CT after 3 months of cobimetinib therapy shows disease regression **(C)**. Control ^18^FDG-PET/CT after 6 months of cobimetinib therapy confirms CMR **(D)**. The femoral biopsy sample shows fibrosis in the intratrabecular space and foamy histiocytes (hematoxylin eosin x100) **(E)**. Femur biopsy with Touton giant cells (arrow) and foamy histiocytes (arrow) (hematoxylin eosin) **(F)**.

In 2019, the patient had already been evaluated for a fever of unknown origin and maculopapular skin rash. At that time, he had reported persistent complaints for the past 3 years. Repeated routine diagnostic tests were performed, and confirmed central diabetes insipidus, significant right-sided carotid artery stenosis, and preocclusive stenosis of the renal artery. The unusual association of these rare symptoms has been attributed to a possible histiocytic disease and an ^18^FDG-PET/CT scan supported the diagnosis with sclerotic lesions in the metaphysis of the long bones and cutaneous/skeletal involvement was also described (**Figure 2A**). A biopsy of the right femur had been performed, which confirmed BRAF-negative ECD (**Figures 2E, F**). A brain MRI confirmed CNS involvement. Based on the patient’s history he had advanced heart disease, which was verified by a CT scan and echocardiography. A pathologic role of ECD was suspected for this. In March 2021, treatment with pegylated interferon alpha was started at a dose of 180 ug once a week but was ineffective based on response assessment with ^18^FDG-PET/CT in June 2021 (**Figure 2B**). Due to the ineffectiveness of interferon-alpha treatment, cobimetinib therapy was initiated at a dose of 60 mg once daily. After 6 months of treatment, in December 2021, ^18^FDG-PET/CT showed CMR (**Figure 2C**). During cobimetinib therapy, the patient developed skin symptoms in the form of a maculopapular rash, and dose reduction was required after the first month. The skin symptoms resolved on the modified dose (40 mg once daily), and the patient is still in CMR (**Figure 2D**). With continued use of cobimetinib, the patient was able to work again and return to his daily life.

### Case 3

**Figure 3:**
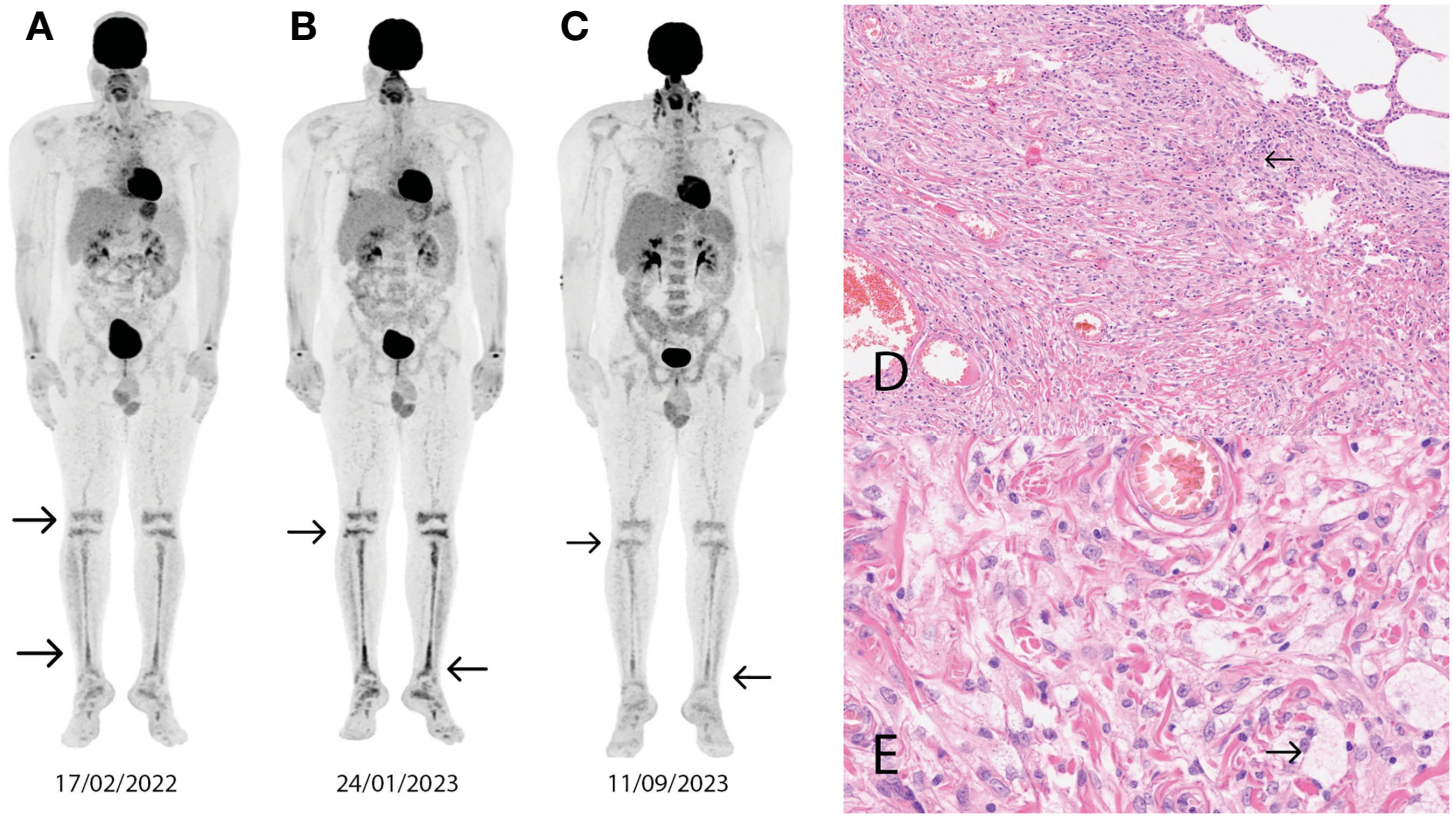
Abnormal contrast uptake (arrows) on ^18^FDG-PET/CT image in the lower extremities (SUVmax 4.4-7.2) **(A)**. Control ^18^FDG-PET/CT image after interferon alpha administration shows refractory disease (SUVmax 7.4-9.4) **(B)**. Control 18FDG-PET/CT image after 3 months of cobimetinib administration shows disease regression (SUVmax 3.2) **(C)**. Pulmonary tissue sample shows fibrosis with foamy histiocytes and lymphoplasmocytic infiltration (arrow) (hematoxylin eosin x100) **(D)**. Pulmonary tissue sample shows fibrosis with foamy histiocytes (arrow) and lymphoplasmocytic infiltration (hematoxylin eosin x400) **(E)**.

**Figure 4:**
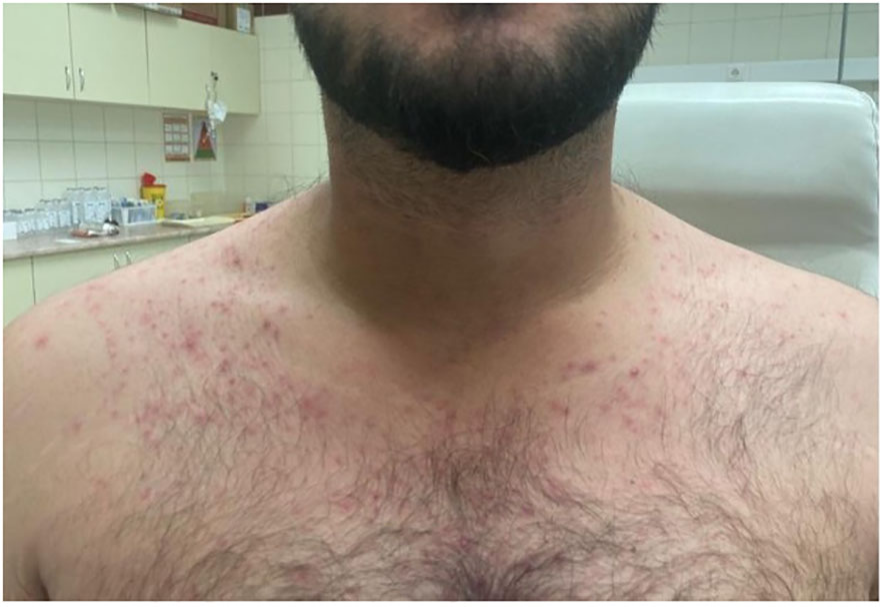
A maculopapular rash appeared after less than two weeks of cobimetinib therapy.

In 2016, a 28-year-old male patient with no significant medical history presented with difficulty breathing. Blood levels of electrolytes, glucose, liver, and kidney function tests, in addition to levels of LDH and inflammatory markers, were normal. A chest CT scan showed pulmonary fibrosis, and the possibility of interstitial lung disease was considered. In 2017, a histologic sample from the lung confirmed BRAF mutation-negative ECD. The patient was followed up regularly without any intervention until 2022. In 2022, skin symptoms (erythema and papules) appeared. The first hematologic consultation was in January 2022, where routine laboratory tests showed elevated creatinine kinase (CK) and LDH levels. Staging with ^18^FDG-PET/CT was performed and showed skeletal involvement in the lower extremities (**Figure 3A**). Femoral bone biopsy supported the diagnosis of ECD in 2017 (**Figures 3C–E**). A brain MRI showed no signs of CNS involvement. A CT scan and echocardiography did not confirm cardiac involvement. Considering the bone, skin, and pulmonary involvement, we started the treatment in November 2022. In the first line, we administered interferon-alpha therapy, but no significant improvement was detected on the control ^18^FDG-PET/CT scan in February 2023 (**Figure 3B**). In the second line, we initiated cobimetinib therapy at a dose of 60 mg once daily. As an adverse event, maculopapular rash occurred during the first two weeks of treatment (**Figure 4**), and a dose reduction (40 mg once daily) was necessary. After three months of cobimetinib therapy, control ^18^FDG-PET/CT showed a partial metabolic response (PMR) with the moderated dose, and we also observed an improvement in disease-related symptoms.

## Discussion

ECD is a rare disease and its diagnosis is often challenging for clinicians due to its wide range of symptoms. One of the most important diagnostic tools is whole-body ^18^FDG-PET/CT, which is able to identify characteristic bilateral and symmetric osteosclerosis of the diaphyseal and metaphyseal parts of the long bones. ^18^FDG-PET/CT is useful not only for diagnosis, but also for response assessment and follow-up of these patients during and after treatment. In addition to ^18^FDG-PET/CT, MR imaging is the best technique for the detection and characterization of cardiac, pericardial, CNS, and renal abnormalities (15–17). In the case of small but aggressive lesions, the detection threshold of PET/CT may be a limitation of the diagnostic capability. In these cases, the role of MRI scans becomes more important. In addition to radiological findings, it is highly recommended to confirm the diagnosis with a tissue biopsy and to perform mutation analysis for further therapeutic decisions. Reverse hybridization was carried out using the BRAF 600/601 StripAssay according to the manufacturer’s protocol (ViennaLab Diagnostics, Vienna, Austria). For BRAF-negative cases, a next-generation sequencing (NGS)-based detection platform was utilized. NGS analysis was also performed according to the manufacturer’s protocol (Illumina, San Diego, CA, USA). Identification of phospho-Erk status was not available.

There are not any specific laboratory findings that help in the diagnosis of ECD but CRP and interleukin-6 (IL-6) elevation can be seen on some occasions and also specific organ involvement can result in laboratory abnormalities (2). The lack of data pertaining to the exact levels of inflammatory markers is a limitation of this case study, although the CRP, IL-6, and TNF-alfa levels could indicate disease activity.

The management of ECD has evolved in the past decade. In BRAF-positive patients, we recommend first-line treatment with BRAF inhibitors, which are associated with quick and sustained responses. According to the LOVE study, the response rate to BRAF inhibitor regimens (e.g. vemurafenib and dabrafenib) was 91% in BRAF mutation-positive patients (18). In the majority of cases, the therapeutic response to BRAF inhibitor monotherapy is favorable, but there are some examples in the literature where patients did not respond to therapy. In these cases, the combined administration of BRAF and MEK inhibitors may be an option, but there is a greater chance of developing side effects, such as arthralgia, fatigue, or heart failure (19, 20). Considering the higher toxicity of vemurafenib based on the literature (20, 21), dabrafenib was chosen in our case at a dose of 75 mg twice daily and therapy resulted in CMR. Guidelines usually suggest a dose of 150 mg twice daily but given the higher risk of side effects and the great therapeutic response, we decided not to administer the full dose. However, there is no evidence of comparative efficacy or tolerability between any of the BRAF inhibitors. In BRAF mutation-negative patients the first-line treatment option in our clinic is interferon alpha, but the therapeutic responses are not satisfactory. This observation differs from the data in the literature. Based on the study by Braiteh et al., a great therapeutic response was observed in 3 cases as early as 1 month after the start of interferon-alpha treatment (22). In another study Hervier et al. observed no improvement with low-dose interferon-alpha, but a favorable response was seen with increased-dose treatment over a longer period of time (23). According to Arnaud et al. the 5-year survival rate with interferon alpha is approximately 68% (8). For these patients, second-line MEK inhibitor therapy (cobimetinib, trametinib) should be recommended, based on the excellent response rates observed in a published case series and a prospective clinical trial (24). With second-line cobimetinib, both of our BRAF mutation-negative patients showed a satisfactory therapeutic response by ^18^FDG-PET/CT, but early dose reduction was necessary due to dermatologic adverse events. In Hungary, the medication allowance is made by the National Institute of Pharmacy and Nutrition and financed by the Hungarian National Health Insurance Fund.

Two of our patients had central diabetes insipidus, requiring endocrinology consultation, which underlines the importance of a multidisciplinary approach to ECD patients. Desmopressin nasal spray was effective in both cases.

According to the study by Cohen et al. 75% of patients relapsed within 6 months after discontinuation of BRAF inhibitor therapy, as confirmed by ^18^FDG-PET/CT. In two cases, progressive CRP elevation preceded clinical symptoms. The resumption of BRAF inhibitor treatment yielded favorable outcomes for all participants (18). Reiner et al. studied 22 patients with histiocytic neoplasms and they found that seventeen (77%) patients relapsed after interruption of targeted therapy, and the median relapse-free-survival rate was 8.52 months (25).

Our patients reported a significant improvement in quality of life after starting targeted therapy, and with moderate doses of MEK inhibitors side effects did not affect their daily lives.

## Conclusion

Based on our experience, the diagnosis and treatment of ECD patients require a multidisciplinary approach. New targeted treatments have improved the quality of life and survival of patients with ECD (18). Treatments with BRAF and MEK inhibitors are associated with significant and long-term responses with an acceptable safety profile. In the event of adverse events, dose reductions can be made without loss of efficacy. However, many patients still favor classic systemic therapies, which are administered for a limited period of time with the potential for cure, while targeted agents require prolonged therapy. The possibilities of planned treatment breaks and the durability of therapy-free remissions should be explored to guide individualized therapies. Although the effectiveness of BRAFi and MEKi therapy in ECD is known, considering the rarity of the disease and the small number of cases reported in the literature, any clinical data could contribute to a better understanding of the disease and the right therapeutic decision.

## Data availability statement

The original contributions presented in the study are included in the article/**Supplementary Material**. Further inquiries can be directed to the corresponding author.

## Ethics statement

Ethical approval was not required for the study involving humans in accordance with the local legislation and institutional requirements. Written informed consent to participate in this study was not required from the participants or the participants’ legal guardians/next of kin in accordance with the national legislation and the institutional requirements. Written informed consent was obtained from the individual(s) for the publication of any potentially identifiable images or data included in this article.

## Author contributions

AGu: Writing – original draft. LIP: Writing – original draft. JM: Writing – review & editing. EV: Writing – review & editing. JB: Writing – review & editing. JT: Writing – review & editing. SB: Writing – review & editing. ZH: Writing – review & editing. ILS: Writing – review & editing. AGa: Writing – review & editing. ÁI: Writing – review & editing. FM: Writing – review & editing.
